# Comparison of Reaction Response Time between Hand and Foot Controlled Devices in Simulated Microsurgical Testing

**DOI:** 10.1155/2014/769296

**Published:** 2014-07-06

**Authors:** Marcel Pfister, Jaw-Chyng L. Lue, Francisco R. Stefanini, Paulo Falabella, Laurie Dustin, Michael J. Koss, Mark S. Humayun

**Affiliations:** ^1^Doheny Eye Institute, 1450 San Pablo Street, Los Angeles, CA 90033, USA; ^2^Department of Ophthalmology, Keck School of Medicine, University of Southern California, 1450 San Pablo Street, Los Angeles, CA 90033, USA; ^3^Department of Ophthalmology and Visual Sciences, Federal University of Sao Paulo (UNIFESP), Rua Botucatu, No. 740, 04023-900 Sao Paulo, SP, Brazil; ^4^Department of Preventive Medicine, University of Southern California, 1975 Zonal Avenue, Los Angeles, CA 90089, USA; ^5^Department of Ophthalmology, Ruprecht-Karls-University, Grabengasse 1, 69117 Heidelberg, Germany

## Abstract

*Purpose*. We hypothesized that reaction times (RTs) for a switch release are faster for hand-controlled than for foot-controlled switches for physiological and anatomical reasons (e.g., nerve conduction speed). The risk of accidental trauma could be reduced if the surgeon reacted quicker and therefore improve the surgical outcome.* Method*. We included 47 medical professionals at USC. Demographics and handedness were recorded. Under a microscope, a simple reaction time test was performed, testing all extremities multiple times in a random order. Additionally, a subjective questionnaire was administered. *Results*. The mean RTs for hands are 318.24 ms ± 51.13 and feet 328.69 ± 48.70. The comparison of hand versus foot showed significant shorter RTs for the hand (*P* = 0.025). Partially significant differences between and within the experience level groups could be demonstrated by level of education (LE) and microscopic surgeries/week (MSW) (*P* = 0.57–0.02). In the subjective questionnaire, 91.5% (*n* = 43/47) of test subjects prefer to use hand controls. *Conclusion*. Our data show that the RT for hands is faster than feet. Similarly the subjective questionnaire showed a greater preference for hand actuation. This data suggest a hand-controlled ophthalmic instrument might have distinct advantages; however, clinical correlation is required.

## 1. Introduction

Very delicate eye surgeries are usually performed by a surgeon, working through a microscope, who is often required to make quick intraoperative decisions. The surgical management of vitreous and retinal pathologies (e.g., retinal detachment or vitreous bleeding) can include removal of the vitreous, intervention on the retina, or intraocular illumination and magnification. Vitreoretinal surgeries are dynamic and precise maneuvers that require a fast reaction time (RT). The RT represents the time between the initiation of a given event (e.g., intraocular bleeding) and the surgeon's response to that event (e.g., elevation of intraocular pressure) [1]. A short RT might be associated with better outcomes and with the prevention of iatrogenic trauma.

All currently commercially available vitrectomy systems are controlled using a foot-pedal (e.g., Stellaris PC—Bausch & Lomb; Constellation Vision System—Alcon; Eva—DORC; NovitreX—Oertli). When the pedal is depressed, the console machine responds by increasing the vacuum and cut rate at the tip of the vitreous cutter. If the retina is inadvertently sucked into the cutter, the surgeon must react quickly to release the pedal, thereby stopping or reducing the suction force. By doing so, the surgeon can prevent serious complications and damage to the retina, such as retinal detachment, retinal holes, or vitreous hemorrhage. Any of these events can cause severe long-term retinal tissue damage with a possible need for further surgery or may even result in permanent vision loss. The length of the release time (pedal) plays a key role in the outcome.

Several articles have been published on simple RT tests [2], but only a few studies have measured eye-hand or eye-foot response times [2, 3]. The hypothesis of the present study was that for physiological, anatomical (e.g., nerve-conduction velocity), and ergonomic reasons, the time required to release a switch with the hand is shorter than the time required to release a switch with the foot. If this hypothesis is correct, then the risk of trauma to the eye could, in theory, be reduced if the cut and vacuum rates were controlled by hand and if so, then the results of the surgery might be improved.

Our study was designed to evaluate the RTs of surgeons or future surgeons, testing both the dominant and nondominant hands and feet. To the best of our knowledge, this is the first RT study in which medical professionals were tested while using a microscope. Data analysis included the subjects' age, gender, medical training, frequency of surgery performed (with and without microscope), and participation in extracurricular activities involving a hand switch. In addition, we measured the intrinsic RT for a mechanical switch release. The collected data could reinforce the need for a hand-controlled vitrectomy system.

## 2. Materials and Methods

A total of 47 volunteers, all medical students and ophthalmic surgeons from the Department of Ophthalmology at the Keck School of Medicine of the University of Southern California, participated in this study. Written informed consent to participate in this study was obtained from all subjects. The protocol was approved by the University of Southern California Health Science Institutional Review Boards (IRB) at IRB Submission Tracking and Review system (iStar) number HS-13-00467. All participants were at least 18 years of age on the day of their participation. Exclusion criteria included active injury affecting any extremity or self-report of an unresolved concussion.

The subjects' age, sex, dominant hand, years of medical training (student-consultant), experience with a surgical microscope, and participation in extracurricular activities involving use of a hand switch (e.g., computer gaming/playing a musical instrument) were recorded. The dominant hand was assessed by the questionnaire. To determine the participants dominant foot, we performed the so-called “Kick-Test” for each participant in order to determine the dominant foot. This information was gathered using an anonymous multiple choice questionnaire completed by the participant directly before the RT testing. Each test subject was assigned to a number for data anonymization.

After the test, participants were asked two subjective questions about the use of hand and foot switches: (1) which extremity did you feel reacted the fastest? and (2) which extremity did you feel the most comfortable using?

## 3. Test Equipment and Setup

Each test subject was placed in the following setup to mimic a real eye surgical environment. The participant was seated at a table with a preinstalled surgical microscope (Nikon SMZ-645 StereoZoom microscope with adjustable holder from Diagnostic Instrument). To prevent unwanted distraction, the test room was kept quiet, the lights were dimmed, and only the examiners were present [4, 5]. The subject was asked to look into the surgical microscope, under which both a red and a green light emitting diode (LEDs) were placed. The red LED would turn on to indicate that the experiment had started and that the subject should press and hold the switch and wait for the green LED to appear. The green LED would light randomly, between 2 and 15 seconds after the beginning of the test, to prevent predictability. Each of the four extremities of each subject was tested five times. The mean value of these five tests was used as the reaction time of a participant extremity. The order in which the extremities were tested was randomized. Based on previous publications, the cut off for RTs recorded was set to 180 ms (minimum) and 500 ms (maximum) [2, 6].

The interval between the start of the experiment (red LED) and the green LED lit time was pseudorandomly programmed in a CPU board (6 external interrupts control, 40 MHz CPU on R-Engine-A board time counters of 0.6 *μ*s time resolution from Tern Inc.) with a custom C++ program. The RT was defined as the time between the lighting of the green LED and the moment the electrical break of the switch circuit happened. The green LED signal and the electrical break signal were automatically acquired by the programmable board, and the difference was calculated and stored. In the program, a RT longer than 0.5 second was considered abnormal and, therefore, was excluded automatically. Release of the snap switch before the green LED signal was also considered a failed trial.

**Figure 1:**
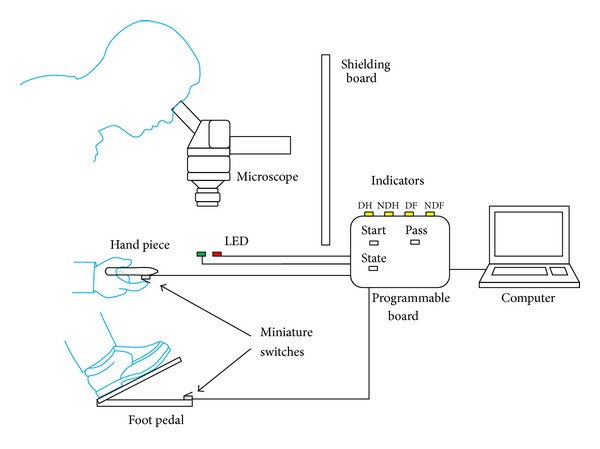
Schematics of systematic setup for the response time experiment.

To accurately evaluate the RT from human test subjects and minimize the machine's RT, we considered the moment of the electrical break of a pressed subminiature snap switch (D2F-FL with lever, Omron Electronics Inc.) as the onset of the human response. The RT of this subminiature snap switch is less than 1 ms. The RT was examined using a high-speed video camera (640 × 512 resolution at 1000 fps, MotionScope M1, RedLake). For hand tests, we used a dummy hand piece mounted with the D2F subminiature snap switch (Figure 1). For foot tests, we used a conventional foot pedal (BL2390, Stellaris PC foot pedal, Bausch & Lomb) with the same D2F subminiature snap switch mounted underneath the foot pedal. The testing with the high-speed camera was performed 10 times for the hand switch and foot switch with the original test equipment and analyzed by J.-C. L.

## 4. Statistical Methods

The statistical significance for within subject differences in RTs was assessed using paired *t*-tests. Analysis of variance tests were used to compare RTs between subcategories, adjusting for age and gender. The covariate adjusted means and standard errors are reported in the tables. Trend tests were also run across ordinal categories. For comparison of RTs by gender, the analysis of covariance was adjusted for age, and the analysis by age group was adjusted for gender. SAS V9.3 programming language (SAS Inst., Cary, NC) was used for all analysis, and the accepted level of significance for all tests was *P* < 0.05.

## 5. Results

As we tested and compared dominant versus nondominant hands and feet, we could demonstrate significantly faster RTs with the dominant extremity (*P* > 0.011; Tables 1 and 2).

The results of this simple RT test demonstrate significantly faster reaction times of the hands compared to the feet (*P* < 0.01; Table 3).

Male subjects were significantly faster with both hands and feet than were female subjects (Table 4).

The results of the subjective questions were in favor of the handheld instruments.

We could demonstrate a trend toward slower RTs for the hands with increasing age of the subjects. Statistically significant differences of the RTs for the feet could be demonstrated when comparing the different age groups (DF *P* = 0.004; NDF *P* = 0.01), but no statistical trend could be shown (DF *P* = 0.68; NDF *P* = 0.51) (Table 7).

Except for DH and NDH difference across education levels (test for trend *P* = 0.03), no statistical significance and no trend could be demonstrated by analyzing the subjects' different experience levels or number of surgeries performed per week (Tables 8 and 9).

As a variable of daily routines we chose the frequency of computer gaming and home-row typing, based upon statements in the questionnaires. For computer gaming frequency, significance was found for DH-NDH (test for trend *P* = 0.01) and NDF (*P* = 0.02). All other tests were not significant (Tables 10 and 11).

The results of the machine RT testing (hand switch versus foot switch) showed for the mini-joystick an average bouncing time of 8.75 ms ± 1 ms. For the foot pedal, the bouncing time was 64.1 ms, with standard deviation of 24.4 ms.

## 6. Discussion

We hypothesized that RTs with the hand are shorter than RTs with the foot, which could be demonstrated as a proof of principle with this cohort of medical students, physicians in training, and fully trained specialists. It is, however, important to note that a good surgical result depends on many more factors than RT. The RT of a surgeon is objectively measurable, whereas the essential surgical setting of experience balanced with a well-trained operating room team is at least as important. Since possible variations in the design of new surgical devices may facilitate faster RTs, we wanted to understand how these changes might be reflected among different age groups, genders, and training levels.

Many changes in navigation tools and surgical devices have been introduced over the last few decades. In the early 1990s, the semiautomatic transmission vehicle was introduced in the Formula 1 car racing game. In a sport where high performance is crucial in every aspect, the hand-controlled pedal shift had completely displaced the conventional gearbox with the foot-controlled clutch within just 5 years. Changes can also be seen in aviation. With advancing technology, the fly-by-wire system, a computer-assisted navigation unit, became more and more common in commercial and military airplanes. The centerpiece control unit for the pilot is a multifunctional hand-controlled sidestick.

Interestingly, in ocular surgery, a highly delicate and individual medical specialization, some basic handling steps have not changed in decades. The use of foot-pedal controls can be tracked to the time when the suction and ultrasound force for phacoemulsification [7] were introduced in 1967. Foot-pedal control of the cut rate and suction force for pars plana vitrectomy [8] was introduced in 1972. With rapid technological advances, new inventions in robotics and microdevices have become available, providing an opportunity to fundamentally rethink and update surgical systems that were developed in the 1960s and 1970s. This has already happened in fields such as neurosurgery, with the use of live MRI imaging or robot-assisted surgical tools.

To determine if the benefit is dependent on age, sex, experience, or level of education, we created a questionnaire for our participants to analyze if there exists significant differences among subgroups. We could demonstrate that the average RT for hands was significantly faster than the average RT for feet (*P* = 0.025). This reflects our expectation of a shorter nerve-conduction time for the brain-hand combination than for the brain-foot combination because the neural pathway from the brain to the hand is shorter. The dominant hand and the dominant foot, as determined by a kick-test, were significantly faster than the nondominant side (hands *P* = 0.01; feet *P* = 0.01). Also, more than 91% of all participants felt more comfortable with and preferred the hand switch, as they stated in the questionnaire (see Tables 5 and 6).

Consistent with previous simple RT studies, the RTs of male participants were significantly faster than those of female participants with all four extremities. (*P* = 0.001–0.005) [9–11]. Again, it is necessary to mention that a faster RT does not imply a better surgeon or a better surgical outcome. When analyzing the subgroups with regard to their experience level, we found no statistically significant faster RTs in more advanced or better educated participants (Table 8). However, the results showed a close to significant trend toward shorter RTs for the dominant hand. This shows again the complexity of factors that lead to a good surgery, as it might be reasonable to assume that the surgical skills of the more experienced surgeon and the surgical outcomes achieved by this surgeon would be better. With regard to the subjective questions, it is also interesting to note that experienced surgeons (fellows and practitioners), even when they were well trained with the foot switch, stated that they felt faster and more comfortable with the hand switch (87.5% preferred the hand switch) (see Tables 5 and 6). We assume that this is an indicator that the hand is the human's favorite tool and the training effect for other extremities is limited due to physiological conditions (e.g., nerve-conduction velocity).

When we sorted the participants by age, those between 20 and 35 years old had significantly faster RTs with the dominant hand and foot than those who were 44 years of age or older. This finding is also reflected in previous studies that found a lengthening of RTs beginning in the late 20 s [11, 12]. RTs for the feet were especially faster in the younger cohort of this study (Table 7). Based on this finding, we conclude that hand-controlled devices could have a positive effect for older surgeons, even though they are more experienced. Also RTs become more variable in older test subjects [13].

We found no significant difference in RTs to be associated with the frequency with which the participants performed surgery using a surgical microscope (Table 9). This perhaps can be linked to basic shortcomings of the questionnaire used in studies. Although we tried to formulate clear multiple choice questions, the participants' answers remain subjective. Thus, a complete verification of the given answers is impossible.

To get an impression of the individual backgrounds of the participants in our study, we tried to acquire information about the subjects' everyday activities, such as home-row typing, computer gaming, or playing a musical instrument. None of these items showed positive correlations to the RTs.

To further investigate the methodological bias we measured, in addition to the RTs of medical professionals' extremities, the RTs for a miniature hand joystick switch (10 kOhm pot joystick potentiometer, rotation angle: 50°, 254 series, CTS Electronic Components) and a conventional foot switch (BL2390, Stellaris PC foot pedal, Bausch & Lomb). In the conventional foot switch, the effective aspiration range is controlled by the pedal's travel distance from the triggering position to the furthest down position. The state-of-the-art machine does provide programming options for users to modify the vacuum levels of the beginning and ending positions across the entire effective travel range. The lowest vacuum that can be programmed at the beginning is zero. In this case, the aspiration at the cutter tip can increase as the user pedal is pressed down further past the triggering position.

A timely response to some medical trauma situations, such as a sudden bleeding in the eye or a clog in a vitreous cutter, requires a prompt cessation of the aspiration power. In the ideal case, assuming the user takes no time to retract the foot, the pedal will still take some time to bounce back to the initial position with zero aspiration. With this time element in mind, we measured the bouncing time of a miniature joystick and a foot pedal. The bouncing time is defined as the traveling time from its far most position to its resting position after the switch is suddenly released. For the mini-joystick, the bouncing time is 3 ms ± 1 ms. If there is a knob with mass of 0.677 g ± 0.001 g for ergonomic purposes, the bouncing time is 8.75 ms ± 1 ms. For the foot pedal, the bouncing time is around 64.1 ms, with standard deviation of 24.4 ms. As a consequence, even in an ideal case in which the user can retract his/her extremity instantly, the machine will still take a certain time to return to zero aspiration. This result strongly suggests that a hand joystick switch is preferred to reduce the RT that can cause unwanted medical trauma.

In our setup, the participants saw a red LED as a sign that the experiment had started. We are aware that this red LED could be interpreted as a warning sign that an event is likely to occur in the near future. It is reported that subjects react faster when they are given a warning indication [14]. However, we do not believe the LED lights provided any additional bias to our results. A number of unintended events/outcomes can occur during ophthalmic surgery (e.g., retinal bleeding, retinal breaks, inadvertent suction of the retina, retinal incarceration, etc.) which require immediate reaction by the surgeon. Therefore, while performing surgical procedures, ophthalmic surgeons are already highly concentrated, focused, and aware of unexpected events that could occur. We also focused on the central visual field with a small LED on which the subjects had to concentrate and to which they had to react. It is known that signals in peripheral visual fields are correlated to slower RTs [14].

Further, the complete release of the switch and the full stop of the cutter that are relevant in vitreoretinal surgery procedures. The variation and dynamic control of the cut rate and vacuum also play a critical role in ophthalmic surgery. Since the hands are a human's most precise tool and since hands have higher tactile acuity than feet, it is likely that the control of the cutter by a hand switch is safer and, therefore, more favorable.

A possible shortcoming of this study was that we tried to simulate a real surgical setting as well as possible, but the test was still performed in an artificial environment. This might have affected the results of the participants as it is likely that the level of awareness is higher in the operating room when performing actual surgery on a human patient. Furthermore, we used an original foot pedal for vitreoretinal surgery (Bausch & Lomb) and a one pressure-point, no dynamic prototype switch as a hand piece. Based on our findings, we suggest further studies with an advanced hand piece and with more specific questions in the census questionnaire. The use of more subgroups and a larger sample size should also be considered.

In spite of these shortcomings, we believe that we demonstrated objective findings in this study: primarily the superior RT of the hand versus the foot. This finding could be an incentive for a transition in device design by medical companies and could also be the basis for a new approach in education for the next generation of surgeons.

## 7. Conclusion

This research contributes to our understanding of the average RT of medical professionals (i.e., ophthalmologists at different levels of training) when they are faced with a specific event happening through a surgical microscope. We chose the RT as an easy-to-test objective parameter that might influence the surgical outcome. We could demonstrate that the RT with hands was significantly faster than with the foot. Experience and level of education had no significant influence on the reaction time. Also, advantages of the hand switch can be seen for the handheld mini-joystick as the bouncing time was only 8.75 ms ± 1 ms compared to 64.1 ms ± 24.4 ms for the foot pedal. We feel that our findings can contribute to future approaches in the design of surgical instruments not only in ophthalmology but also in other fields. To improve RTs in the surgical field, hand-controlled devices appear to be desirable. Further studies are needed.

## Figures and Tables

**Table 1:** Dominant versus nondominant hand (*n* = 47 subjects); *P* < 0.05 = statistically significant.

	Mean ± SD (ms)	Paired *t*-test/*P* value
Dominant Hand (DH)	311.1 ± 51.9	
Nondominant Hand (NDH)	325.4 ± 56.8	
Difference (dominant/nondominant)	−14.3 ± 37.3	**T** = −**2.63**, **d** **f** = **46** **P** = **0.011**
Effect size	0.383	

**Table 2:** Dominant versus nondominant foot (*n* = 47 subjects); *P* < 0.05 = statistically significant.

	Mean ± SD (ms)	Paired *t*-test/*P* value
Dominant foot (DF)	321.5 ± 51.0	
Nondominant foot (NDF)	335.9 ± 53.0	
Difference (dominant/nondominant)	−14.4 ± 36.5	**T** = −**2.32**, **d** **f** = **46** **P** =** 0.01**
Effect size	0.395	

**Table 3:** RTs of hands (average) versus feet (average) (*n* = 47 subjects); *P* < 0.05 = statistically significant.

	Mean ± SD (ms)	Paired *t*-test/*P* value
Hands (average of dominant and nondominant hand)	318.2 ± 51.1	
Feet (average of dominant and nondominant foot)	328.7 ± 48.7	
Hands versus feet Difference (hands-feet)	−10.4 ± 30.9	**T** = −2.32, **d** **f** = 46 **P** = 0.025
Effect size	0.337	

**Table 4:** RT analysis of variance of gender (mean ± SE); *P* < 0.05 = statistically significant.

	n	DH (ms)	NDH (ms)	Difference (ms)	DF (ms)	NDF (ms)	Difference (ms)
Female	21	338 ± 10	354 ± 11	−16 ± 8	345 ± 10	360 ± 11	−15 ± 8
Male	26	289 ± 9	302 ± 10	−13 ± 7	302 ± 9	316 ± 10	−14 ± 7
Difference		49	52	3	43	44	1
Effect size		1.068	1.026	0.083	0.938	0.867	0.028
*F*-test, 1 df		13.31	11.24	0.05	9.10	8.79	0.01
ANOVA *P*		**<0.001**	**0.002**	0.83	**0.004**	**0.005**	0.93

**Table 5:** Subjective question 1: percentage of preferred extremity.

Which extremity did you feel reacted the best?			
	Hand	Foot	No difference
All participants *n* = 47	43/47 (91,48%)	2/47 (4,25%)	2/47 (4,25%)
Experience surgeons (fellows/practitioners) *n* = 24	21/24 (87,5%)	1/24 (4,16%)	2/24 (8,33%)

**Table 6:** Subjective question 2: percentage of preferred extremity.

Which extremity did feel most comfortable using?			
	Hand	Foot	No difference
All participants *n* = 47	43/47 (91,48%)	1/47 (2,12%)	3/47 (6,38%)
Experience surgeons (fellows/practitioners) *n* = 24	21/24 (87,5%)	0/24 (0%)	3/24 (12,5%)

**Table 7:** RT analysis of variance of age (mean ± SE).

	n	DH (ms)	NDH (ms)	Difference (ms)	DF (ms)	NDF (ms)	Difference (ms)
Age, gender adjusted							
≥20	3	327 ± 26	367 ± 28	−40 ± 22	388 ± 24	408 ± 25	−20 ± 21
21–27	6	285 ± 18	308 ± 20	−23 ± 16	286 ± 17	306 ± 18	−19 ± 15
28–35	22	305 ± 10	322 ± 11	−17 ± 8	324 ± 9	327 ± 9	−3 ± 8
36–43	13	325 ± 12	329 ± 14	−4 ± 11	313 ± 11	347 ± 12	−33±10
44–51	2	341 ± 26	320 ± 35	21 ± 27	353 ± 29	352 ± 31	1 ± 26
>51	1	427	457	−31	424	426	−2
*F*-test, 5 df		2.34	2.84	0.86	4.13	3.48	1.30
ANOVA *P*		**0.06**	0.10	0.52	**0.004**	**0.01**	0.28
Test for trend		**0.03**	0.42	0.11	0.68	0.51	0.73

**Table 8:** RT analysis of variance of education (mean ± SE); *P* < 0.05 = statistically significant.

P < 0.05 = statistically significant	*n*	DH (ms)	NDH (ms)	Difference (ms)	DF (ms)	NDF (ms)	Difference (ms)
Education							
Student	8	328 ± 19	314 ± 22	14 ± 15	335 ± 20	350 ± 22	−16 ± 16
Resident	14	316 ± 12	326 ± 14	−10 ± 10	319 ± 12	333 ± 14	−14 ± 10
Fellow	11	309 ± 14	323 ± 16	−14 ± 11	330 ± 14	331 ± 16	−1 ± 12
Practitioner	13	287 ± 14	322 ± 15	−35 ± 11	299 ± 14	326 ± 15	−26 ± 12
*F*-test, 3 df		1.07	0.08	1.89	1.11	0.24	0.94
ANOVA *P*		0.37	0.97	0.15	0.36	0.87	0.43
Test for trend		0.09	0.94	0.03	0.20	0.48	0.53

**Table 9:** RT analysis of variance of frequency of surgical microscope use (mean ± SE); *P* < 0.05 = statistically significant.

	*n*	DH (ms)	NDH (ms)	Difference (ms)	DF (ms)	NDF (ms)	Difference (ms)
Use of surgical microscope							
No	9	318 ± 18	328 ± 20	−10 ± 12	346 ± 18	351 ± 19	−4 ± 14
Assisting	7	312 ± 19	313 ± 21	−1 ± 14	322 ± 19	338 ± 20	−16 ± 15
Every month	8	305 ± 17	352 ± 19	−47±12	319 ± 17	334 ± 18	−14 ± 14
Every week	19	315 ± 12	322 ± 13	−7 ± 9	313 ± 12	335 ± 13	−21 ± 10
Every day	4	288 ± 24	305 ± 26	−18 ± 18	308 ± 24	309 ± 26	−1 ± 19
*F*-test, 4 df		0.36	0.80	2.23	0.58	0.42	0.35
ANOVA *P*		0.84	0.53	0.08	0.68	0.79	0.85
Test for trend		0.52	0.63	0.92	0.17	0.28	0.76

**Table 10:** RT analysis of variance of computer gaming (mean ± SE); *P* < 0.05 = statistically significant.

P < 0.05 = statistically significant	*n*	DH (ms)	NDH (ms)	Difference (ms)	DF (ms)	NDF (ms)	Difference (ms)
Computer games							
No	28	313 ± 9	318 ± 10	−5 ± 7	323 ± 9	335 ± 9	−11 ± 7
1x/yr	5	294 ± 21	305 ± 23	−11 ± 16	299 ± 21	311 ± 21	−12 ± 17
1x/month	10	312 ± 15	339 ± 16	−27 ± 11	317 ± 15	331 ± 14	−14 ± 12
1x/week	3	300 ± 27	346 ± 29	−46 ± 21	322 ± 27	350 ± 26	−27 ± 22
Daily	1	379	449	−70	420	495	−75
*F*-test, 4 df		0.74	1.93	1.72	1.32	3.20	0.64
ANOVA *P*		0.57	0.13	0.16	0.29	0.02	0.63
Test for trend		0.87	0.06	0.01	0.70	0.26	0.32

**Table 11:** RT analysis of variance of home row typing (mean ± SE); *P* < 0.05 = statistically significant.

	*n*	DH (ms)	NDH (ms)	Difference (ms)	DF (ms)	NDF (ms)	Difference (ms)
Typing-home Row							
No	14	297 ± 13	308 ± 15	−11 ± 11	309 ± 14	335 ± 15	−27 ± 11
1x/week	2	299 ± 32	326 ± 37	−27 ± 27	327 ± 34	322 ± 36	5 ± 26
Daily	31	318 ± 9	333 ± 10	−15 ± 7	327 ± 9	337 ± 10	−10 ± 7
*F*-test, 2 df		0.91	0.86	0.15	0.55	0.08	1.01
ANOVA *P*		0.41	0.43	0.86	0.58	0.92	0.37
Test for trend		0.20	0.19	0.79	0.30	0.94	0.22
